# Enhanced Primary Motor Cortex Astrocyte Calcium Signaling With Motor Learning

**DOI:** 10.1155/np/5571169

**Published:** 2025-12-22

**Authors:** Ragunathan Padmashri, Anna Dunaevsky

**Affiliations:** ^1^ Department of Neurological Sciences, University of Nebraska Medical Center, Omaha, Nebraska, 68198, USA, unmc.edu

**Keywords:** astrocyte, calcium activity, learning, motor cortex

## Abstract

Astrocytes form an integral part of the nervous system and are proposed to modulate neuronal circuits and behavior. The motor cortex plays a key role in the planning and execution of voluntary movements and makes key contributions to motor skill learning. However, whether motor skill learning modulates astrocytic Ca^2+^ signaling in the primary motor cortex (M1) is not known. To understand the role of astrocytes in the M1, we first characterized the Ca^2+^ signaling properties in astrocyte subcompartments in awake mice. We found that the subcompartments exhibited different Ca^2+^ event properties during the no movement periods and locomotion. We then asked whether astrocytic Ca^2+^ signaling in M1 is modulated with the acquisition of a skilled forelimb reaching task. Astrocytes exhibited altered Ca^2+^ event properties at different stages of learning a forelimb reaching task, with early and transient increases in Ca^2+^ event amplitude being the most prominent. These results demonstrate for the first time that, in addition to previously described synaptic plasticity, astrocytic Ca^2+^ signaling is also modified with motor skill learning.

## 1. Introduction

Astrocytes form an integral part of the nervous system architecture and are in continuous bidirectional communication with neurons. The fine processes of astrocytes are in close proximity with synaptic contacts [1, 2], and the synapse association of astrocytes is a dynamic process [3–6] that can be altered by neuronal activity [7–9]. Astrocytes exhibit excitability in the form of intracellular Ca^2+^ concentration increases that are considered a measure of astrocytic activity and are potentially linked to modulation of neuronal function through release of gliotransmitters [10, 11].

Astrocyte Ca^2+^ signals can occur intrinsically [12–15] or in response to neuronal activity [16, 17]. In vivo studies have revealed that astrocytes display Ca^2+^ elevations in response to synaptic activity from sensory and motor stimuli [18–24], by stimulation of long‐range cholinergic [25, 26] and noradrenergic [27–30] projections. Astrocytes display variable Ca^2+^ signaling properties in different subcompartments such as soma, processes, and microdomains (MD) [15, 23, 31–33].

Studies have demonstrated that manipulating astrocytes, including Ca^2+^ signaling, can affect neuronal function, synaptic plasticity, and interfere with learning [29, 34–37]. Motor skill learning results in structural and functional plasticity in the primary motor cortex (M1) [38–43] through an LTP‐like mechanism [39, 40, 43–45]. Reducing astrocytic activity interferes with motor learning and motor learning induced AMPA receptor insertion in M1 [34]. Astrocytic‐specific manipulations in M1 during a motor learning task alter motor learning and execution, as well as dysregulate M1 neuronal activity [36].

Taken together, these studies suggest that astrocytes play an important role in motor learning and that they do so by mechanisms including regulation of Ca^2+^ signals. However, we currently lack an understanding of motor learning induced alterations in M1 astrocytic Ca^2+^ signaling.

Here, we first characterized the Ca^2+^ signaling properties in the astrocyte subcompartments in awake mice during no movement and movement periods. We then asked whether astrocytic Ca^2+^ signaling in M1 is modulated with the acquisition of a skilled motor task. Our results indicate that the subcompartments exhibit different Ca^2+^ event properties during no movement and movement periods. In addition, astrocytes exhibit altered Ca^2+^ event properties in various subcompartments at different stages of learning a forelimb reaching task.

## 2. Materials and Methods

### 2.1. Animals

Mice were cared for in accordance with NIH guidelines for laboratory animal welfare. All protocols were approved by the University of Nebraska Medical Center Institutional Animal Care and Use Committee. Two transgenic mouse lines, a lox‐STOP‐lox‐cytosolic GCaMP6f (B6;129S‐*Gt*(*ROSA*)*26Sor* ^*tm95.1*(*CAGGCaMP6f*)*Hze*^/J, Jax 024105) and GLAST‐CreER (Tg (Slc1a3‐cre/ERT) 1Nat/J, Jax 012586) were crossed to obtain inducible astrocyte‐specific cytosolic GCaMP6f expression (GLAST‐CreER;GCaMP6f mice). Genotyping was performed by PCR using primers, and the double‐transgenic mice were weaned at P21 and treated with Tamoxifen. Tamoxifen was dissolved in corn oil (10 mg/mL) and administered intraperitoneally (i.p.) at 100 mg/kg for 5 days. Male mice were used for the experiments.

### 2.2. Tissue Preparation and Immunohistochemistry

Mice were sacrificed with Tribromomethanol (Avertin, 400 mg/kg i.p.) and transcardially perfused with 4% paraformaldehyde in phosphate buffer (0.1 M) at postnatal day 51, brains then removed and kept overnight in 4% PFA at 4°C. Coronal brain sections (100 µm) were cut on a vibratome in phosphate‐buffered saline (1x PBS). Sections containing the primary motor cortex were selected for immunostaining. For immunohistochemistry, sections were rinsed in PBS (3 × 5 min), incubated for 3 min in 0.3% Triton‐X 100 in PBS, followed by incubation in 0.3% Triton‐X 100% and 5% normal goat serum (NGS) in PBS for 1 h to permeabilize membranes and reduce nonspecific staining. Sections were then incubated with primary antibodies (anti‐GFP, chicken, 1:500, Invitrogen A10262; anti‐S100β, rabbit, 1:100, Abcam ab868) in 0.3% Triton‐X 100% and 5% NGS in PBS overnight at 4°C. The GFP antibody was used to reveal GCaMP6f protein that has a permutated EGFP. The following day, sections were rinsed in PBS (3 × 10 min) and then incubated with secondary antibodies (goat anti‐chicken Alexa Fluor 488, 1:1000, Invitrogen A‐11039; goat anti‐rabbit Alexa Fluor 594, 1:200, Invitrogen A‐11012) for 90 min at room temperature. After further rinsing in PBS (3 × 10 min), sections were mounted with Fluoro‐Gel mounting medium.

### 2.3. Confocal Imaging

Confocal imaging was performed on a Nikon A1R upright microscope, and images of the immunostained sections were acquired using a 20x objective (0.75 NA) for assessing GCaMP6f expression in astrocytes. Images were acquired in the forelimb region of M1 with a resolution of 512 × 512 and image stacks with a step size of 1 µm were collected. Maximum intensity projected images of 10 optical sections were opened in ImageJ, which was used for the analysis of recombination efficiency to determine the proportion of S100β+ cells that are also GFP+ (GCaMP6f+).

### 2.4. Cranial Window Implantation

At postnatal day 30, mice were anesthetized with isoflurane (3% for induction, 1.5%–2% maintenance during surgery) and a cranial window was implanted over the primary motor cortex as previously described [46]. Dexamethasone (0.2 mg/kg) and carprofen (5 mg/kg) were administered 15 min before the surgery to prevent swelling of the brain and to reduce inflammation during the craniotomy. Following anesthesia, eyes were covered with ointment to prevent drying. Head was shaved and skin was wiped with iodine and alcohol swabs. An opening was made in the skin, and the skull above the primary motor cortex was exposed. Skull was scraped with a scalpel and dried with compressed air. A small well was drilled on the left frontal bone, and a mini bolt was screwed into the drilled well. A 5 mm craniotomy centered on bregma was made across the sutures above the primary motor cortex with a dental drill. Upon removal of the skull flap, a piece of Gelfoam soaked in saline was placed over the exposed brain to prevent drying of the brain. Using a beveled glass pipette, a mixture of AAV1.CaMKII.0.4.Cre (1:5000) and AAV1.CAG.FLEX.tdTomato was injected for expression of tdTomato in layer 2/3 neurons in the M1. A volume of 0.2–0.4 µL of the virus solution was injected with a Picospritzer, and injections were performed at a depth of 200–300 μm in 2 sites. Pipettes were left in the brain for 4–5 min after the injection to avoid backflow. The exposed surgery site was then rinsed with enrofloxacin antibiotic solution (0.5 μg/mL) and covered with a 5 mm diameter sterile cover glass that was placed over the opening and permanently glued to the skull with cyanoacrylate‐based glue. The dura remained intact during the procedure. A metal head plate with a circular opening was glued over the cover glass and held with a mixture of dental cement, polyacrylic glue, covering the exposed skull, and the mini bolt screwed into the skull bone to ensure stability of the head plate. Mice were injected with 0.5 mL of sterile saline subcutaneously. Mice were treated with antibiotic enrofloxacin (5 mg/kg) twice daily for 6 days after surgery to prevent bacterial infection and injected once daily with carprofen (5 mg/kg) for 3 weeks following surgery to reduce inflammation. Mice were allowed 3 weeks to recover from the surgery before start of the imaging experiments.

### 2.5. Habituation of Mice for Imaging in Awake Conditions

Mobile home cage (MHC, Neurotar) was used for imaging in awake mice, where a head‐fixed mouse can move around in an air‐lifted MHC that features a flat floor and tangible walls and explore the environment under stress‐free conditions. Prior to the imaging experiments, mice were habituated to the MHC by gradually increasing the duration of the habituation sessions every day and also acclimating the mouse to the sounds of the laser scanning mirrors [46]. The habituation phase was started 2 weeks after the cranial window was implanted.

### 2.6. Electromyography‐Implantation of Electromyography (EMG) Electrodes

Mice were anesthetized with isoflurane (3% for induction, 1.5%–2% maintenance). The forelimb was shaved and skin was wiped with alcohol swabs. The paired fine wire needle electrode (30 mm, 27 ga, Chalgren EMG and EEG electrodes, Motion Lab Systems Inc) was inserted into the triceps brachii muscles, and the needle was then gently retracted to slide it off the wires. The wires were connected to an EMG amplifier (MA‐300 EMG system, Motion Lab Systems, Baton Rouge, LA) before start of the experiment. The voltage output was read by custom LabVIEW (National Instruments) software and sampled at 1.2 kHz by a 16‐bit National Instruments analog‐to‐digital converter. A 60 Hz notch filter and a 20–400 Hz band pass filter were applied to the collected EMG data.

### 2.7. Motor Skill Training

Motor skill training was performed as previously described [43]. Seven‐week‐old mice following cranial window implantation were food‐restricted (85% of their free‐feeding weight). During training, mice were fed food pellets (Dustless Precision Pellets 20 mg, rodent grain‐based diet, Bioserv #F0163) during and after the training session. All training was done in the afternoon to reduce the effects of circadian variations on training. Before food restriction, mice were habituated for 20 min in a Plexiglas box with an attached platform that could be reached through a thin slit on the front wall. The following day, a pretraining session was performed to determine forelimb preference. Pellets were then placed on the side that enabled the use of the preferred forelimb only. Mice were trained to reach through the slit with their preferred forelimb and grasp and retrieve individual food pellets. Mice had one training session per day that lasted 30 min or 100 reaches. Motor skill performance was quantified by the success rate (% of successful retrievals). Motor skill training was performed in 6 mice. One mouse that exhibited a high success rate on day 1 showed no subsequent improvement in the motor skill performance and was excluded from the study.

### 2.8. In Vivo Imaging

All imaging was performed with a multiphoton microscope (Moving Objective Microscope; Sutter Instruments) equipped with a resonant scanner and piezo stage (nPFocus400, nPoint) coupled to a Ti:Sapphire laser (Chameleon Vision II, Coherent). Images were collected with a Nikon water immersion objective (25X, 1.05 NA). Excitation power measured at the back aperture of the objective was typically about 20 mW, and laser power was modulated using a Pockels cell. GCaMP6f and tdTomato were excited at 920 nm, and emission was detected with GaAsP detector (Hamamatsu Photonics) fitted with a 535/50 bandpass filter and 610/75 bandpass filter and separated by a 565 nm dichroic mirror. The tdTomato‐labeled dendrites served as landmarks to reliably return to the same regions and image the same astrocytes over repeated days. ScanImage (v5.1, Vidrio Technologies) software [47] was used for imaging. The EMG data (LabVIEW) and video recordings (acquisition every 0.1 s, ThorCam) were acquired simultaneously with the time‐lapse volume imaging data and synchronized through the resonant scanner trigger. The EMG acquisition was performed on a subset of mice and was not performed on mice that were trained on the forelimb reaching task and on naïve mice that received no motor skill training. Each optical section was collected at 512 by 512 pixel resolution (0.18 µm/pixel), and a 40–50 µm thick volume was acquired at a step size of 1 µm. Time‐lapse imaging was performed every 2 s for a period of 5 min. All the imaging fields were in layer 1 within the forelimb M1 as determined by stereotaxic measurements (between 0.75 and 2 mm lateral to the midline and between 1 mm anterior and 0.5 mm posterior to the bregma; caudal forelimb area [48]. Imaging timepoints (following baseline session for determination of forelimb preference [D0], 2 h after the first training session [D1], and a day after the last training session [D6]) were chosen to examine motor learning induced changes in astrocytic Ca^2+^ signaling (Figure 1A). The contralateral hemisphere and ipsilateral hemisphere to the trained forelimb are abbreviated as CH and IH respectively.. Naïve mice that received no training sessions and for which forelimb preference was not determined were imaged at the same timepoints to examine astrocyte Ca^2+^ signaling over days in untrained mice. Mice were kept on the stage for a maximum of 2 h.

Figure 1Imaging astrocyte Ca^2+^ activity in the primary motor cortex in awake mice. (A) Schematic of experimental design. (B) Confocal image of a coronal brain section containing the M1 from a postnatal 7‐week‐old GLAST‐CreER; GCaMP6f mouse that was injected with 100 mg/kg (i.p.) tamoxifen at 3 weeks for astrocyte‐specific expression of GCaMP6f. Staining with GFP for GCaMP6f (green), S100β (magenta) as an astrocyte marker, and overlay of the two. Expression of cyto‐GCaMP6f was observed in 43% ± 1.5% (*n* = 16 fields from 4 mice) of S‐100β‐labeled astrocytes in the M1. (C) (C1) Co‐occurrence between the onset times for video events and EMG events during movement periods. *R* ^2^ = 0.9997, *p* < 0.0001, 70 movement events from 20 imaging sessions, 3 mice. (C2) Quantification of peak EMG amplitude in EMG only and video events accompanied with EMG events. EMG only: 1.76 ± 0.09 V; video + EMG: 5.33 ± 0.31 V; *p* < 0.0001. *N* = 60 for EMG‐only events and *N* = 69 for video accompanied with EMG events.  ^∗∗∗∗^ *p* < 0.0001. (D) Ca^2+^ activity in a single astrocyte expressing GCaMP6f. Individual representative frames from 5 min imaging experiment with AQuA‐detected events in corresponding time frames. Time frames from no movement periods and movement periods are shown.

**Figure:**
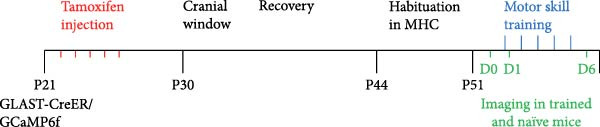
(A)

**Figure:**
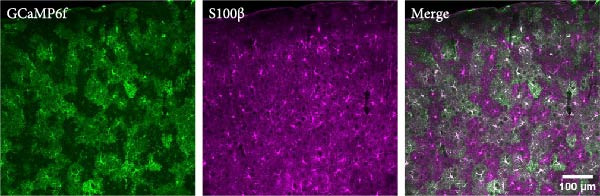
(B)

**Figure:**
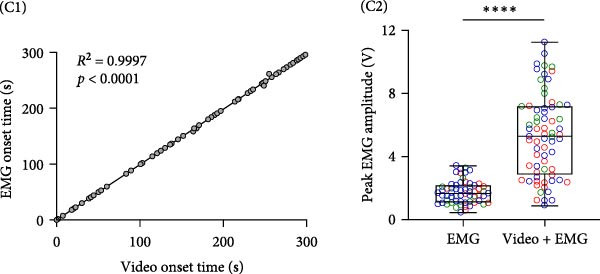
(C)

**Figure:**
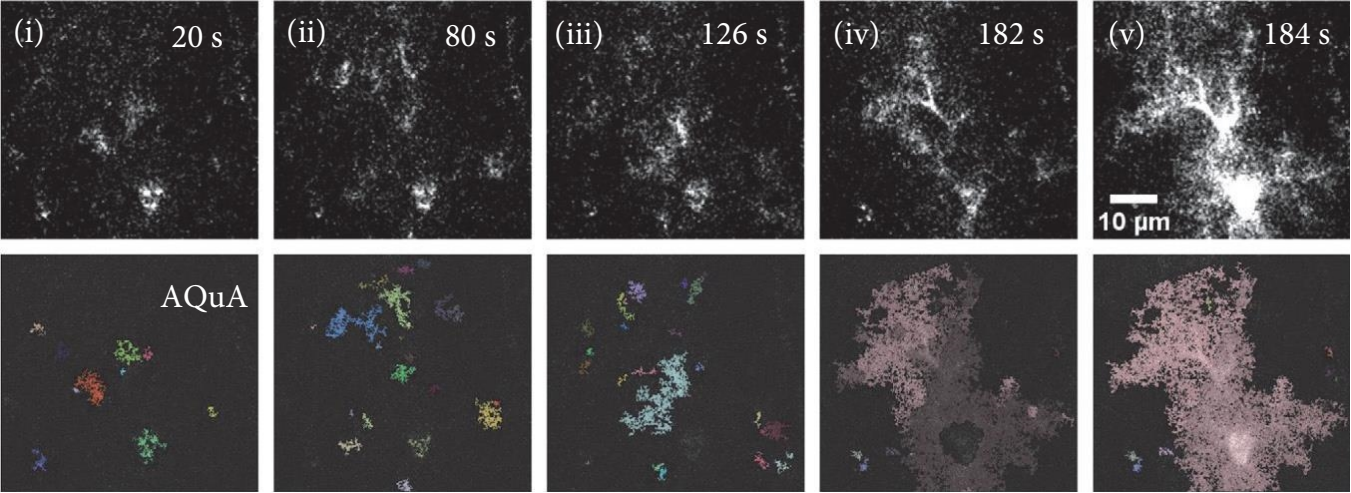
(D)

### 2.9. Categorization of Behavior‐Based EMG Data and Video Recordings

Two‐photon imaging was synchronized with video recording and, in a subset of mice, with EMG recordings. Using custom Matlab (Mathworks) software, the EMG data were extracted, the EMG time series were full‐wave rectified and low‐pass filtered at 475 Hz using a zero‐lag 9th‐order Butterworth filter. The threshold to detect the EMG signal was set at 3 SD values above the baseline (during 20 s of no EMG activity), and Clampfit v10.6 (pCLAMP, Molecular Devices) software was used to detect the onset times and amplitude of the EMG signals.

The video recordings were analyzed in ImageJ, and the entire video time series was manually inspected for periods of rest, locomotion, engaging of forelimbs, and grooming episodes, which were categorized into no movement and movement periods. The analysis for the onset times for these different behaviors was performed prior to the analysis of EMG signals. The video onset times were later correlated to the onset times of the EMG activity. Analysis of the video and EMG recordings revealed that video and EMG events co‐occurred during periods of clearly visible movement, although there were additional EMG events that were not associated with visible movements in the video recordings. Since the onset times for the video and EMG events were highly correlated (Figure 1C), the imaging sessions were categorized into no movement and movement periods based on the video recordings.

### 2.10. Image Analysis

Correction for motion artifacts during the image series was performed using the ImageJ plugin moco [49]. Images were corrected for tdTomato bleed‐through into GCaMP6f (green) channel by quantifying percent bleed‐through and subtracting out the bleed‐through from images of the GCaMP6f images. The images acquired as a volume were maximum intensity *z* projected in 5 µm volumes for further analysis. Event‐based analysis of astrocyte Ca^2+^ image events was performed using Astrocyte Quantitative Analysis (AQuA) software [50]. Individual astrocytic domains were visually identified. Two ROIs were used to delineate areas used for further analysis in each individual cell: one ROI outlining the entire astrocyte for the territory occupied by the cell, and a second ROI outlining the soma that served as a landmark. Any AQuA‐detected event that had a value of zero for the event minimum distance from the landmark was identified as soma or a global event containing the soma. The AQuA‐detected events were categorized into MD (0.5 to less than 5 µm^2^), processes (Proc, 5–100 µm^2^), and soma/global event containing the soma (Soma). Based on the video recordings, the Ca^2+^ events were categorized into events occurring during no movement and movement periods. Furthermore, the Ca^2+^ events that occurred within 4 s prior to a movement episode were not included in the analysis, as these events are likely to be occurring during premovement associated periods.

### 2.11. Statistics

Analysis was done on GraphPad Prism, and summary data are expressed as mean ± SEM. Normal distribution was tested using Shapiro–Wilk normality test. Data were considered significantly different when *p* value was less than 0.05. To test for statistical significance, Mann Whitney test (Figure 1C), Kolmogorov–Smirnov test (Figure 2A,B), repeated measures one‐way ANOVA with Dunnett’s multiple comparisons test (Figure 3A), mixed‐effects ANOVA model with Tukey’s multiple comparisons test (Figures 2C,D and 3B,C), mixed‐effects ANOVA model with Sidak’s multiple comparisons test (Figure S1) were used. Linear regression was performed for co‐occurrence between the onset times for video events and EMG events during movement periods (Figure 1C).

Figure 2Astrocyte Ca^2+^ event properties in the primary motor cortex. (A) Frequency distribution of Ca^2+^ event sizes for microdomains (MD) and processes (Proc) during no movement and movement periods. (B) Frequency distribution of Ca^2+^ event sizes for events that included the soma during no movement and movement periods. (C) Ca^2+^ event properties during no movement periods. Imaging was performed on 3 mice. (C1) Average Ca^2+^ event amplitude, MD: 0.36 ± 0.01, Proc: 0.24 ± 0.01, Soma: 0.29 ± 0.01; MD vs. Proc, *p* < 0.0001; MD vs. Soma, *p* < 0.0001; Proc vs. Soma, *p* = 0.0002; (C2) Average Ca^2+^ event duration, MD: 7.06 ± 0.08 s, Proc: 10.61 ± 0.42 s, Soma: 12.21 ± 0.49 s; MD vs. Proc, *p* < 0.0001; MD vs. Soma, *p* < 0.0001; Proc vs. Soma, *p* = 0.09; (C3) Average Ca^2+^ event rise time to peak, MD: 3.49 ± 0.04 s, Proc: 4.87 ± 0.22 s, Soma: 5.72 ± 0.24 s; MD vs. Proc, *p* < 0.0001; MD vs. Soma, *p* < 0.0001; Proc vs. Soma, *p* = 0.05. (C4) Average Ca^2+^ event decay time, MD: 3.59 ± 0.04 s, Proc: 5.66 ± 0.23 s, Soma: 6.4 ± 0.31 s; MD vs. Proc, *p* < 0.0001; MD vs. Soma, *p* < 0.0001. MD, *N* = 45 cells, Proc, *N* = 45 cells, and Soma, *N* = 40 cells. (D) Ca^2+^ event properties during movement periods. (D1) Average Ca^2+^ event amplitude, MD: 0.4 ± 0.02, Proc: 0.28 ± 0.01, Soma: 0.41 ± 0.04; MD vs. Proc, *p* < 0.0001; MD vs. Soma, *p* = 0.95; Proc vs. Soma, *p* = 0.002. (D2) Average Ca^2+^ event duration, MD: 6.68 ± 0.14 s, Proc: 9.44 ± 0.43 s, Soma: 13.47 ± 0.87 s; MD vs. Proc, *p* < 0.0001; MD vs. Soma, *p* < 0.0001; Proc vs. Soma, *p* = 0.0002. (D3) Average Ca^2+^ event rise time to peak, MD: 3.35 ± 0.09 s, Proc: 4.28 ± 0.24 s, Soma: 5.65 ± 0.44 s; MD vs. Proc, *p* = 0.0003; MD vs. Soma, *p* < 0.0001; Proc vs. Soma, *p* = 0.014. (D4) Average Ca^2+^ event decay, MD: 3.34 ± 0.07 s, Proc: 5.08 ± 0.33 s, Soma: 7.58 ± 0.56 s, MD vs. Proc, *P* < 0.0001; MD vs. Soma, *P* < 0.0001; Proc vs. Soma, *p* = 0.0006. MD, *N* = 30 cells, Proc, *N* = 30 cells, and Soma, *N* = 25 cells. mean ± sem  ^∗^ *p* < 0.05,  ^∗∗^ *p* < 0.01,  ^∗∗∗^ *p* < 0.001,  ^∗∗∗∗^ *p* < 0.0001. (E) Proportion of cells exhibiting the number of soma responses (parathensis) during movement epochs (1–4 M).

**Figure:**
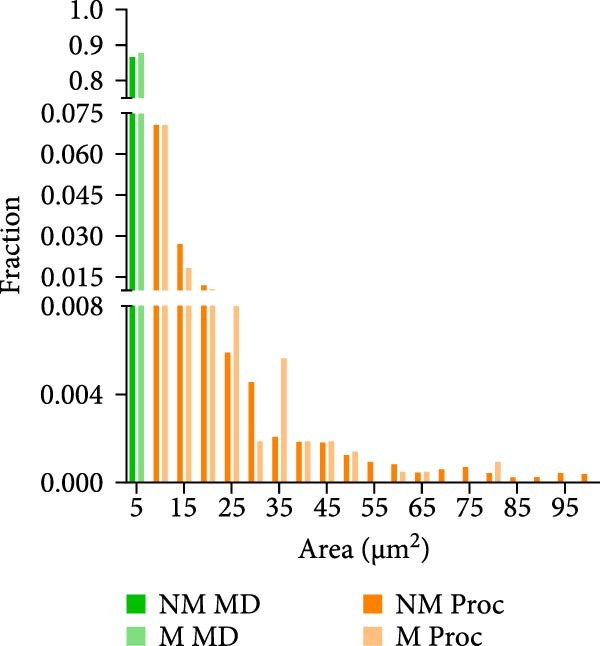
(A)

**Figure:**
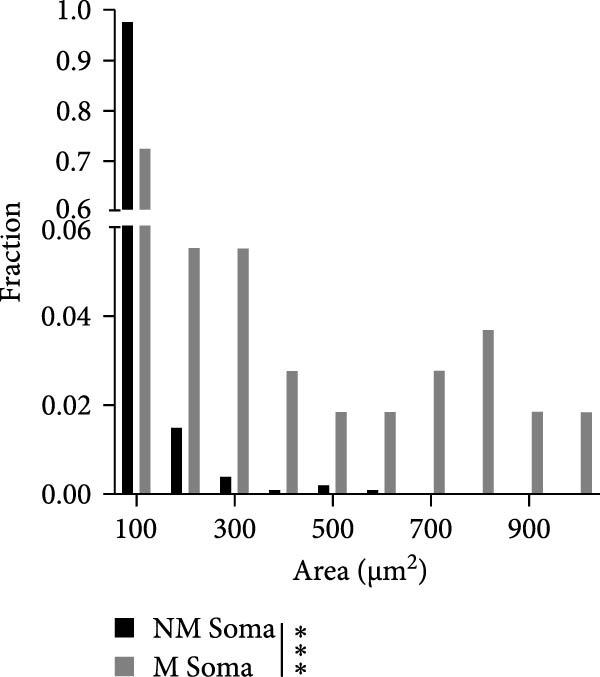
(B)

**Figure:**
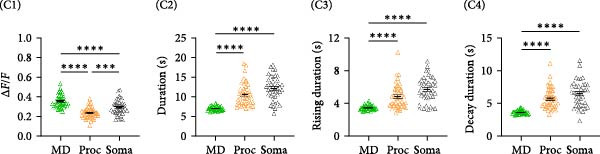
(C)

**Figure:**
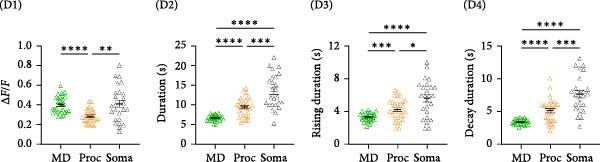
(D)

**Figure:**
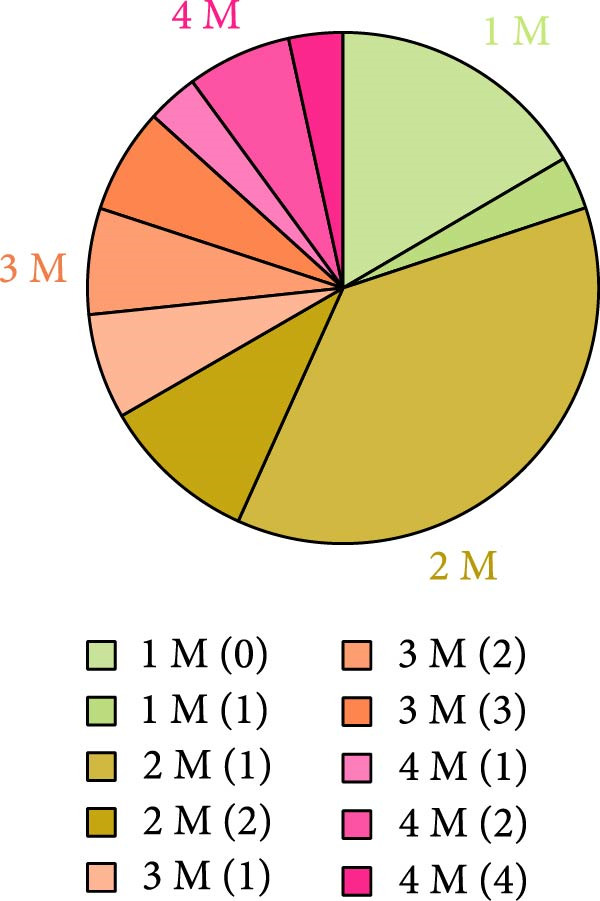
(E)

Figure 3Astrocyte Ca^2+^ activity is increased in the primary motor cortex with motor learning. (A) Success rate of mice trained on forelimb reaching task (*N* = 5 mice). Repeated measures one‐way ANOVA, *F*_(1.998,7.993)_ = 8.964, *p* = 0.009. (B) Event properties of Ca^2+^ activity during no movement periods (B1–B4) in the ipsilateral hemisphere (IH) and contralateral hemisphere (CH) to the trained forelimb for baseline (D0), after 2h of training (D1), and a day after the last training session (D6). Imaging was performed on 5 mice, and analysis was performed for cells from 3 mice for the IH and from 5 mice for the CH, with *N* = 11−22 cells. (B1) Average data for Ca^2+^ event amplitude. MD: Training day x Hemisphere, *F*_(2,110)_ = 6.895, *p* = 0.0015, Proc: Training day x Hemisphere, *F*_(2,71)_ = 3.627, *p* = 0.032 and Soma: Training day x Hemisphere, *F*_(2,89)_ = 10.94, *p* < 0.0001. MD: D0, IH 0.29 ± 0.01, CH 0.34 ± 0.01; IH vs. CH, *p* = 0.003, D1, IH 0.28 ± 0.02, CH 0.42 ± 0.03; IH vs. CH, *p* = 0.0001. Proc: D1, IH 0.18 ± 0.01, CH 0.24 ± 0.02; IH vs. CH, *p* = 0.024. Soma: D1, IH 0.2 ± 0.01, CH 0.33 ± 0.03; IH vs. CH, *p* = 0.0001. (B2) Average data for Ca^2+^ event duration. MD: Training day x Hemisphere, *F*_(2,71)_ = 7.807, *p* = 0.0009, MD: D0, IH 7.08 ± 0.14, CH 7.06 ± 0.12; IH vs. CH, *p* = 0.89, D1, IH: 7.04 ± 0.24s, CH: 6.65 ± 0.18 s; IH vs. CH, *p* = 0.21; D6, IH: 6.86 ± 0.12 s, CH: 7.85 ± 0.29 s; IH vs. CH, *p* = 0.004. (B3) Average data for Ca^2+^ event rising duration. MD: Training day x Hemisphere, *F*_(2,71)_ = 10.51, *p* = 0.0001, Soma: Training day x Hemisphere, *F*_(2,56)_ = 3.57, *p* = 0.035. MD: D6. IH 3.31 ± 0.05, CH 4.07 ± 0.22; *p* = 0.003; Soma: D6, IH 5.26 ± 0.46, CH 6.4 ± 0.28; *p* = 0.045. (B4) Average data for Ca^2+^ event decay duration. MD: Training day x Hemisphere, *F*_(2,71)_ = 1.925, *p* = 0.15. MD: D6, IH: 3.59*s* ± 0.08, CH: 3.79 ± 0.15s, *p* = 0.25. (C) Ca^2+^ event properties during movement periods in the IH and CH to the trained forelimb for baseline (D0), after 2 h of training (D1), and a day after the last training session (D6). (C1) Average data for Ca^2+^ event amplitude, (C2) event duration, (C3) event rising duration, and (C4) event decay duration in MD, Proc, and Soma are shown. Ca^2+^ event amplitude, MD: Training day x Hemisphere, *F*_(2,52)_ = 10.87, *p* = 0.0001. Proc: Training day x Hemisphere, *F*_(2,50)_ = 4.127,*p* = 0.02. Soma: Training day x Hemisphere, *F*_(2,42)_ = 6.293, *p* = 0.004. MD: D1, IH 0.29 ± 0.02, CH 0.45 ± 0.03; IH vs. CH, *p* < 0.0001. Proc: IH 0.2 ± 0.01, CH 0.28 ± 0.02; IH vs. CH, *p* = 0.004. Soma: D1, IH 0.21 ± 0.02, CH 0.36 ± 0.03; IH vs. CH,*p* = 0.0002. mean ± sem,  ^∗^ *p* < 0.05,  ^∗∗^ *p* < 0.01,  ^∗∗∗^ *p* < 0.001,  ^∗∗∗∗^ *p* < 0.0001.

**Figure:**
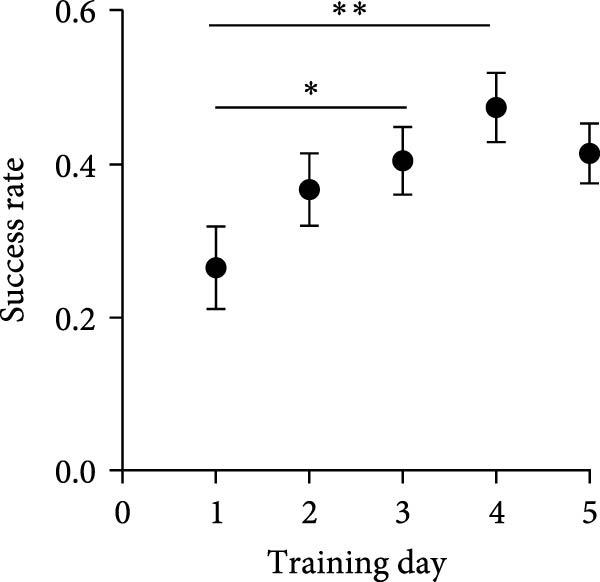
(A)

**Figure:**
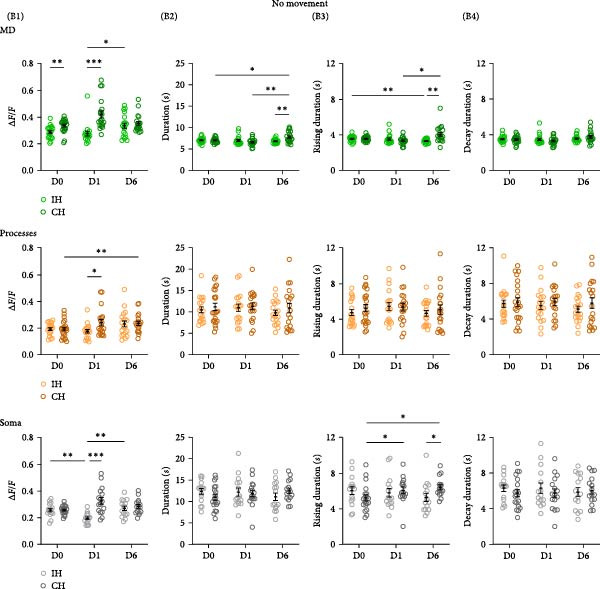
(B)

**Figure:**
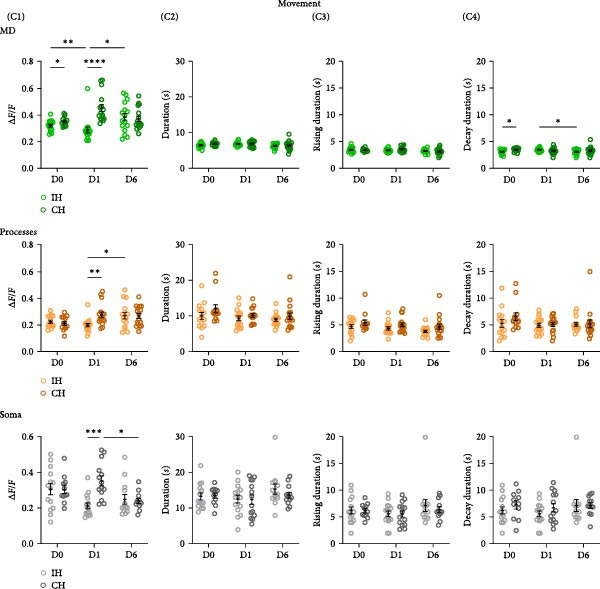
(C)

## 3. Results

### 3.1. Astrocyte Ca^2+^ Activity in the Primary Motor Cortex in Awake Mice

To study astrocyte Ca^2+^ signaling in vivo, we bred Rosa26‐lsl‐GCaMP6f mice to GLAST‐CreER mice to express GCaMP6f specifically in astrocytes, and expression was induced by injecting tamoxifen (100 mg/kg for 5 days, P22−26, Figure 1A) i.p. 3 weeks after tamoxifen injections, expression of cyto‐GCaMP6f was observed in 43% ± 1.5% S‐100β labeled astrocytes in the M1 (Figure 1B). We implanted a cranial window above M1 and performed volumetric two‐photon imaging in awake, head‐fixed mice that were free to move or rest in an airlifted MHC. The imaging sessions were categorized into no movement and movement periods based on the synchronized video recordings and, in some cases, on EMG recordings (Figure 1C). To understand which subcompartments were active during the no movement and movement periods, we focused the analysis on single cells in layer 1 of M1 (Figure 1D). We performed the analysis of Ca^2+^ activity on single GCaMP6f‐expressing astrocytes using AQuA software that performs event‐based analysis of Ca^2+^ events.

Ca^2+^ signals were detected in the soma as well as in the branches of the astrocytes (Figure 1D). Analysis of the distribution of the non‐soma events revealed that the majority were small events (<5 µm^2^) with a few events being larger than 50 microns (Figure 2A). We therefore divided the non‐soma containing events into two subcompartments, MD (0.5–5 µm^2^) and processes (5–100 µm^2^). With movement, no differences were observed for the distribution of the area of the events in the MD and processes (Figure 2A). However, as shown for other brain regions, in M1, movement often resulted in global Ca^2+^ events that involved a large portion of the astrocyte territory, including the soma (Figure 1D). Analysis of calcium events encompassing the soma showed that there was a significant right shift in the distribution of event sizes during movement episodes (Figure 2B, *p* = 0.0006). Analysis of the Ca^2+^ event properties for each subcompartment during no movement (Figure 2C) and movement periods (Figure 2D) revealed significant differences. During no movement periods, MD events had a larger mean amplitude (Δ*F*/*F*, Figure 2C1), exhibited shorter durations (Figure 2C2), and showed faster rise time to peak and decay time (Figure 2C3 and C4) than Ca^2+^ events in the processes and soma. Similar differences in Ca^2+^ parameters between subcompartments were observed during movement periods (Figure 2D). Moreover, there was a significant increase in the amplitude of the Ca^2+^ events in MD, processes and soma compared to the event amplitude observed during resting periods (MD No movement vs. MD Movement, *p* = 0.028; Process No movement vs. Proc Movement, *p* = 0.003; Soma No movement vs. Soma Movement, *p* = 0.005).

We also observed that not all astrocytes had a soma containing response to movement at all times. To interrogate this further, we determined the proportion of responding cells. The number of movement epochs within an imaging session ranged from 0 to 4. For each cell that was imaged during a movement bout, we quantified the proportion of times that a soma response was associated with movement bout. We observed that five out of the six cells that were analyzed in imaging sessions with a single movement epoch (1 M in Figure 2E) did not exhibit movement‐induced Ca^2+^ increases in the soma. Interestingly, the same cells exhibited soma Ca^2+^ events during no movement periods. When the animal ran twice or more during an imaging session (2–4 M), astrocytes responded with a soma Ca^2+^ event either during every movement epoch or only in some movement epochs. We found that the majority of the cells exhibited a soma response in a subset of movement episodes (Figure 2E). Moreover, the same movement episode did not elicit a response in every cell that was analyzed within the imaging session, suggesting a heterogeneity in the astrocyte soma response to locomotion.

### 3.2. Astrocyte Ca^2+^ Activity in the Primary Motor Cortex is Modulated With Motor Skill Learning

We earlier showed that reducing astrocytic activity interferes with motor learning and motor learning induced AMPA receptor insertion [34], demonstrating that normal astrocytic activity is necessary for motor learning. To understand whether astrocytic Ca^2+^ signaling in M1 is modulated by the acquisition of a skilled motor task, we performed repeated in vivo imaging of astrocytes in the forelimb area of M1 with motor learning. Mice were trained on a forelimb reaching motor task over 5 days and showed progressive improvement in the rate of successfully retrieved food pellets (Figure 3A). The forelimb area of M1 was imaged repeatedly, first following baseline session for determination of forelimb preference (D0), and following the first (after 2 h of training, D1) and final (a day after the last training session, D6) training sessions. These timepoints were chosen based on earlier studies that showed that motor skill training induces a rapid but transient increase in dendritic spines, accumulation of AMPARs, promotes synaptic plasticity at excitatory synapses, and strengthens synaptic connections in the CH to the trained forelimb [38, 41, 43, 51, 52]. We imaged both the CH and the IH to the trained forelimb.

We examined the properties of astrocyte Ca^2+^ activity in the M1 associated with motor skill learning, during the no movement and movement periods (Figure 3). During no movement periods, significant motor‐skill training induced changes in the Ca^2+^ event amplitude (Figure 3B1) were observed, in the MD, processes, and soma. Interestingly, a significant difference, 17% higher in the CH (*p* = 0.003), was observed between the IH and CH in the baseline session for MD Ca^2+^ event amplitude. This interhemisphere difference in amplitude in MD further increased after 1 day of training, reaching 50% (*p* = 0.0001). However, after completion of training (D6) interhemisphere difference was no longer observed, mainly because of an increase in the amplitude of the IH. In the processes, a 33% increase (*p* = 0.024) was observed in the CH only after 1 day of training. While in the baseline session, no significant interhemisphere differences were observed for Ca^2+^ event amplitude in the soma, a 65% increase (*p* = 0.0001) was observed in the CH after 1 day of training. As with the MDs, the interhemisphere difference was transient and no longer observed at D6. Overall, these results identify a transient increase in Ca^2+^ event amplitude that occurs early in the motor skill training.

We next examined the temporal characteristics of the astrocyte Ca^2+^ events with motor skill training. Learning induced changes in the Ca^2+^ event duration (Figure 3B2) were observed in the MD. Specifically, an interhemisphere difference with longer event duration (14%, *p* = 0.004) of MD in the CH was observed after completion of training (D6). Interestingly, no differences between IH and CH were observed for the duration of soma and processes events following D1 of training or after completion of training. The increase in event duration in the MD was driven by changes in rising duration. The events in MDs and soma showed slower rise time to peak in the CH after completion of training (Figure 3B3). Overall, these results indicated that motor skill learning results in delayed change in the kinetics of the astrocyte Ca^2+^ events.

Analysis of Ca^2+^ event properties during movement periods showed similar enhancement of Ca^2+^ signaling with motor skill learning. Most consistently, a significant but transient increase in Ca^2+^ event amplitude was observed in MD, processes, and soma after 1 day of training (Figure 3C1).

To exclude the possibility that the changes in Ca^2+^ signaling were independent of motor skill training, the forelimb area of M1 was imaged repeatedly in naïve, untrained mice at the same imaging intervals as the trained mice. Naïve, untrained mice exhibited no differences in MD, processes, or soma Ca^2+^ event properties during no movement periods and movement periods (Figure S1A,B).

## 4. Discussion

The goal of the study was to determine whether motor skill training modulates astrocytic Ca^2+^ activity in the primary motor cortex. As the Ca^2+^ activity in the different subcellular compartments in the forelimb area of the M1 in awake mice has not been previously characterized, we first implemented in vivo multiphoton imaging of GCaMP6f expressing astrocytes in awake head‐fixed mice performing unprompted movements in an air‐lifted mobile homecage. To understand the wide range of Ca^2+^ signaling that occurs in the subcompartments, we performed volume imaging that enabled to capture a larger portion of the astrocyte structure. Most previous studies have used ROI‐based analysis on astrocytic subcompartments [13, 15, 23, 53] that use static ROIs. In our study, analysis was performed using AQuA, an event‐based approach where Ca^2+^ events are recognized as distinct events in time that are not spatially restricted [50].

During no movement periods, astrocytes displayed Ca^2+^ activity in the subcompartments and exhibited different Ca^2+^ event properties in the MD, processes, and soma. Notably, MD events showed larger amplitudes, shorter durations, faster rise, and decay times than the events in the soma and processes. This is consistent with previous studies in the somatosensory cortex that have shown that MD and processes display larger amplitudes than soma and exhibit faster durations than soma during spontaneous Ca^2+^ events [13, 23]. While Ca^2+^ events with faster durations (<1.5 s) have been observed [33], others have not observed these fast events in the subcompartments [13, 15, 23, 32]. In our study, the detection of very fast events was limited by the acquisition rates used.

The majority of the cell compartments responded to self‐initiated movements. Ca^2+^ events encompassed most of the astrocyte structure, resulting in a global event when the mouse transitioned from no movement periods to active locomotion. MD and soma events exhibited larger amplitude than processes. While MD events displayed shorter durations, faster rise and decay times, soma events exhibited slower kinetics with longer durations, slower rise, and decay times. We found that not all movement episodes within an imaging session elicited Ca^2+^ responses in the astrocyte soma in M1. Failures in locomotion‐induced Ca^2+^ responses have also been reported in Bergmann glia [28]. Moreover, astrocytic Ca^2+^ responses to pairs of consecutive locomotion episodes have been shown to diminish the response to the second locomotion [28, 54], suggesting astrocytic refractoriness could arise from distinct mechanisms underlying the generation of Ca^2+^ signal.

Perturbation of astrocytes either by selectively attenuating IP_3_R2‐mediated astrocyte Ca^2+^ signaling or using an astrocyte‐specific metabolic inhibitor results in impaired motor skill learning, LTP in slices, and motor learning induced AMPAR insertion [34], showing that normal astrocytic Ca^2+^ signaling is necessary for motor skill learning. Astrocyte‐specific manipulations in M1 during a lever push task alters motor learning and execution, as well as the underlying neuronal population coding [36]. Cortical astrocytes were shown to contribute to motor learning via astrocyte glutamate transporter 1 and calcium regulation. These studies show that astrocytes have a crucial role in motor learning, and that they do so by mechanisms that include Ca^2+^ signaling. However, how learning affects astrocyte Ca^2+^ signaling is not well understood. In a study that examined astrocyte activity with cue‐reward association learning paradigm, it was demonstrated that astrocyte Ca^2+^ responses evolve over the learning period [37]. However, in this study, the activity of the population of astrocytes was examined with fiber photometry, and changes to the activity in different astrocyte subcompartments were not evaluated. Given that the astrocyte function is associated with motor learning, we asked whether astrocytic Ca^2+^ signaling in M1 is modulated with the acquisition of a skilled motor task. We did not image astrocyte Ca^2+^ signaling while the mouse was performing the forelimb task, but rather we quantified astrocyte Ca^2+^ event properties in the different subcompartments at different stages of learning a forelimb reaching task. We found increases in Ca^2+^ event amplitude in the MD and Soma in the CH immediately after training. The understanding of the role of astrocyte Ca^2+^ signaling is an actively debated question [11], and our finding of its modulation with motor skill learning opens further questions. Interestingly, the most pronounced effects of motor skill training on astrocyte Ca^2+^ signaling were found at a time when there is also an increase in the formation of new spines in the CH [40, 43]. In addition, longer event duration in the MD was observed in the CH after completion of training. This increase in duration of the MD Ca^2+^ events occurs at a time when synaptic strengthening and spine AMPAR accumulation in the CH was previously observed [38, 40, 43, 52, 55]. Interestingly, motor skill performance has been shown to be retained days after completion of the training task [45]. It would be interesting to determine if training‐induced modulation of astrocyte Ca^2+^ signaling also persists. Astrocyte Ca^2+^ dynamics changes were mostly observed in the hemisphere contralateral to the trained forelimb, consistent with the lateralized synaptic changes shown by multiple studies [38, 40, 41, 43, 52, 55]. A role for astrocytic perisynaptic processes in regulating dendritic spine stability has been shown earlier [8, 9]. Activity‐dependent enhancement in the motility of perisynaptic astrocytic processes increases synaptic coverage and synaptic stability [8, 9] and was found to be dependent on astrocytic Ca^2+^ signals [9]. Altered dendritic spine stability was shown in dendrites in close proximity to human‐derived astrocytes that exhibited changes in Ca^2+^ signaling [56]. In our study, the altered spontaneous (no movement) Ca^2+^ event properties were notably observed in MD, structures that are known to be tightly apposed to synapses. However, the present study does not capture data to assess the relationship between the altered Ca^2+^ dynamics observed in the astrocyte subcompartments and synaptic changes during the different stages of learning. It is possible that the altered astrocytic Ca^2+^ signaling that occurs during the early and late stages of motor learning could be contributing to the synaptic changes reported in earlier studies. Astrocytes are likely to carry out a widespread role in learning induced synaptic changes, as each astrocyte has the potential to influence thousands of synapses simultaneously.

In summary, our study provides insights into the nature of astrocyte Ca^2+^ signaling in the primary motor cortex and the alterations to the signaling that occur with motor learning, particularly in terms of subcellular astrocyte compartmentation.

## 5. Conclusion

Although it is known that astrocytes respond to behaviorally induced neuronal activity by altering their Ca^2+^ signaling, the dynamics and plasticity of these events in the different astrocyte cellular compartments in the primary motor cortex are not known. We demonstrate that motor skill learning induces changes in astrocytic Ca^2+^ signaling in the primary motor cortex with a temporal and cell compartment specificity.

## Conflicts of Interest

The authors declare no conflicts of interest.

## Funding

This work was supported by the National Institute of Mental Health (R21 MH107029) (to Anna Dunaevsky) and the National Institute of Neurological Disorders and Stroke (R01NS109381) (Anna Dunaevsky).

## Supporting Information

Additional supporting information can be found online in the Supporting Information section.

## Supporting information


**Supporting Informationno movement** Figure S1: Astrocyte Ca^2+^ responses in M1 during repeated imaging. Repeated imaging of the forelimb area of M1 in naïve untrained mice (4 mice) at the same imaging intervals as the trained mice to examine the properties of astrocytic Ca^2+^ events over days. Forelimb preference was not determined and data from both hemispheres were pooled. (A) Event properties for Ca^2+^ activity during no movement episodes (i–iv) is shown. Average data for Ca^2+^ event amplitude, duration, rise time to peak and decay time in MD (D0: *N* = 16 cells; D1: *N* = 13 cells; D6: *N* = 14 cells), Proc (D0: *N* = 16 cells; D1: *N* = 13 cells; D6: *N* = 13 cells), and soma (D0: *N* = 5 cells; D1: *N* = 3 cells; D6: *N* = 5 cells). (B) Event properties for Ca^2+^ activity during movement episodes (i–iv) is shown. Average data for Ca^2+^ event amplitude, duration, rise time to peak and decay time in MD (D0: *N* = 17 cells; D1: *N* = 15 cells; D6: *N* = 14 cells), Proc (D0: *N* = 16 cells; D1: *N* = 14 cells; D6: *N* = 13 cells), and soma (D0: *N* = 14 cells; D1: *N* = 9 cells; D6: *N* = 11 cells). Mean ± sem.

## Data Availability

Data will be made available upon request to the corresponding author.
