# Supplementary figures and images for: Enhanced Primary Motor Cortex Astrocyte Calcium Signaling With Motor Learning
Source: Neural Plast. 2025 Dec 22;2025:5571169. doi: 10.1155/np/5571169 (PMC12767412; doi:10.1155/np/5571169)

## Slide 1
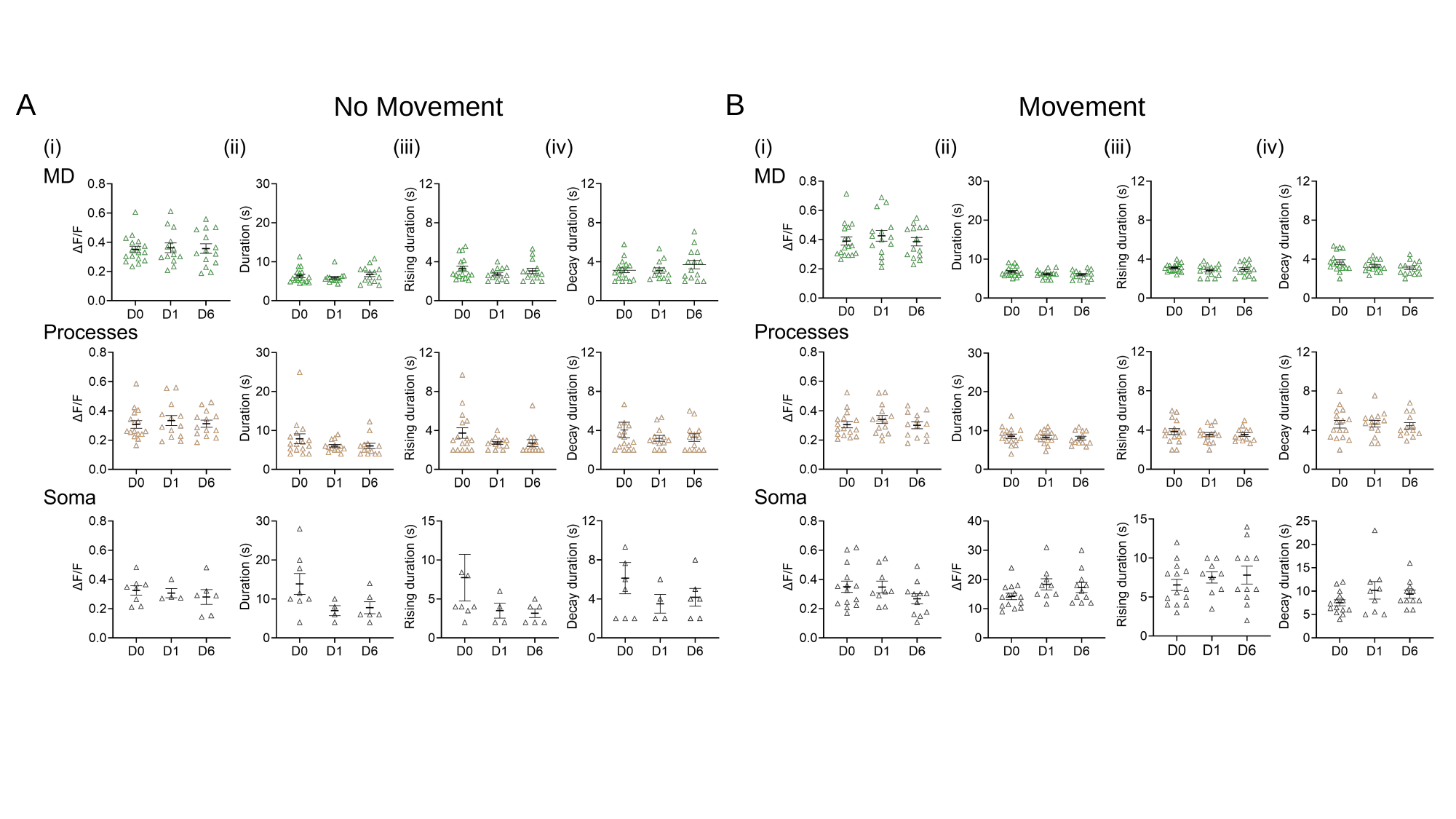

A
B
No Movement
Movement

Supplement: Supplementary file 1 — Supporting Informationno movement Figure S1: Astrocyte Ca2+ responses in M1 during repeated imaging. Repeated imaging of the forelimb area of M1 in naïve untrained mice (4 mice) at the same imaging intervals as the trained mice to examine the properties of astrocytic Ca2+ events over days. Forelimb preference was not determined and data from both hemispheres were pooled. (A) Event properties for Ca2+ activity during no movement episodes (i–iv) is shown. Average data for Ca2+ event amplitude, duration, rise time to peak and decay time in MD (D0: N = 16 cells; D1: N = 13 cells; D6: N = 14 cells), Proc (D0: N = 16 cells; D1: N = 13 cells; D6: N = 13 cells), and soma (D0: N = 5 cells; D1: N = 3 cells; D6: N = 5 cells). (B) Event properties for Ca2+ activity during movement episodes (i–iv) is shown. Average data for Ca2+ event amplitude, duration, rise time to peak and decay time in MD (D0: N = 17 cells; D1: N = 15 cells; D6: N = 14 cells), Proc (D0: N = 16 cells; D1: N = 14 cells; D6: N = 13 cells), and soma (D0: N = 14 cells; D1: N = 9 cells; D6: N = 11 cells). Mean ± sem. [file NP-2025-5571169-s001.pptx]
